# Case of Strangulated Intestine, from Rotation of the Sigmoid Flexure—with Remarks

**Published:** 1843-08

**Authors:** Jacob Bigelow


*Case of Strangulated Intestine, from Rotation of the Sigmoid Flexure—with remarks.*—By Jacob Bigelow, M.D. The Hon. Hugh S. Legare, Attorney-general of the United States, arrived in Boston on Friday, June 16th, and although fatigued by a hasty journey from Washington, was well enough to make calls on some friends in the evening. At 1

o'clock in the night he was seized with frequent abdominal pains, resembling those of colic, and called Dr. Thomas, of Washington, then lodging in the same hotel, to his assistance. During the remainder of the night, and the whole of the next day and night, he was affected with pains alternating with intervals of ease, without constitutional disturbance, and agreeing in character with those of previous attacks to which he had been liable for more than two years, the last occurring in March preceding. Various laxatives and enemata were resorted to, together with counter-irritants, but without removal of the constipation and pain.

Early on Sunday morning I was called to meet Dr. Thomas at Mr. L's lodgings at the Tremont House. I found him then suffering frequent paroxysms of pain, which he referred mostly to the lower abdomen, without distinction of side, but which sometimes mounted above the umbilicus. The pulse was at this time 60, the skin natural, with no tenderness on deep pressure of the abdomen in any part, no meteorism, no nausea. Opiates and other remedies were proposed to him, but declined, on the ground that laxatives and mechanical means had relieved his former attacks. During the morning two doses of Epsom salt, with infusion of senna and tincture of hyoscyamus were given, with frequent enemata both aqueous and stimulating, without effect. The pains did not increase, but a troublesome degree of tenesmus made it necessary to suspend the enemata. Elastic tubes were passed throughout the rectum, and water injected through them in the manner recommended by Dr. O'Beirne, but they could not be carried into the sigmoid flexure. His strength meanwhile remained good and his general condition stationary.

At 6 P. M. he was removed without difficulty to the house of a friend, where he was immediately put into a warm bath of 106 deg., from which he expressed great relief and satisfaction. He was then put to bed, and 60 drops of laudanum were administered in two doses. In about an hour, the relief not being perfect, 40 drops of Munn's elixir of opium were given, soon after which he fell into a quiet sleep, and so remained for about 3 hours. Conditional directions were given for repeating the opiate, but it was not found necessary till near morning, when he took 20 drops of the elixir and slept an hour or two more. On Monday morning at 5 o'clock, I found him more comfortable than before, skin temperate, pulse 64, abdomen not tender but beginning to be tympanitic. Castor oil and senna, with hyoscyamus, were now given and retained, and enemata, fomentations and sinapisms, were resumed as before. The pain did not return with the same severity as before, but meteorism rapidly increased, with restlessness and tenderness on pressure. At 9 A. M. the pulse was 80, and before 12 it was 100. The face of things having become very serious, Dr. Thomas being absent from the city, I requested further consultation, and Dr. Warren was called in. The abdomen was freely leeched and rubbed with croton oil. Various ineffectual attempts were made to overcome the obstruction of the intestine by the introduction of various tubes, by inflation of the rectum with a bellows, and by the tobacco injection administered twice. Under this last remedy he said he felt excited, was stronger but more agitated, and his pulse rose from 130 to 140, with increased force. Each injection contained half a drachm in infusion, and was retained nearly half an hour without narcotism or prostration. During the night the patient was restless, retaining his muscular strength in a considerable degree, and frequently getting up to the close stool in the belief of an approaching evacuation. There was never any vomiting nor nausea; the mind was clear, and the natural decisive tone of voice continued. He complained occasionally of a sense of burning at the epigastrium and upper abdomen. About half an hour before death he got up without assistance, and on lying down asked urgently for water. On receiving it, he pushed it away, saying it was filled with ants. A white paper was then shown him, to which he applied the same remark. On being told it was an illusion of sight, he put forth his hand for the glass, but missed it, said a few words incoherently, leaned back, and expired quietly at half past 5.

*Autopsy seven hours after death.*—Externally the limbs were very rigid, and there was much lividity about the head and back. The abdomen was greatly distended. On laying it open the cavity seemed nearly filled by the sigmoid flexure of the large intestine, which extended across the abdomen into the right hypochondrium, and was in a state of such distension, that its external circumference was in one place fifteen inches. It had a dusky green color, as if from commencing gangrene, but there seemed to be no softening, nor diminution of the natural polish. The two extremities of the flexture connected with the colon above, and rectum below, were felt to be twisted together about the mesentery as an axis, into a firm cord or neck, about an inch in diameter; and on being carefully untwisted, the whole included portion was found to have made four turns, or two entire revolutions upon itself. There was no line of demarcation between the healthy and strangulated portions, nor was there any appearance externally of old disease about this part. The small intestine and the colon were moderately distended, but the rectum was rather contracted. The cavity of the peritoneum contained a small quantity of turbid reddish fluid, and in one place there was recent lymph upon the small intestine, but there were no other appearances of inflammation. Owing to the state of the body and the place of examination, the intestine was not opened, and no farther dissection made.

*Remarks.*—Internal strangulation, we have reason to believe, is a fatal disease except in rare instances in which a spontaneous restoration of the parts may under favorable circumstances have taken place. But the resources of art are for the most part unavailing, from our ignorance at the time, of the nature and place of the lesion, and from the inaccessible situation of the part, unless by a dangerous operation, not to be justified under any diagnosis which can be seasonably made out. Among the various causes known to have occasioned strangulation, the rotation or twisting of the intestine is less common than some others. Yet in addition to the case which has now been described, two others have occurred in this city, under the observation of Drs. Homans and J. B. S. Jackson, the record of which I have seen, in which fatal strangulation occurred from the torsion or twisting of the sigmoid flexure.

Professor Rokitansky, of Vienna, in a work on internal strangulations of the intestines, divides these lesions into three species. Of these, the second species consists in the rotation of one part round an axis most commonly formed by some other part. It appears to be the result of his experience that rotation round the mesentery as an axis can happen only to the small intestine.* But it appears from the case above detailed, and the two others alluded to, that the large intestine is capable of undergoing this rotation, and from its anatomical position, no part seems more exposed to this change of situation than the sigmoid flexure.

From remarks made by Mr. Legare during his illness, it is believed that in some of his former attacks of colic and constipation, relief was obtained by the introduction of the elastic tube beyond the seat of the stricture. This happy result is to be ascribed to the spasmodic character of the obstruction then existing. But when the intestine is rendered impervious by mechanical strangulation, it is evident that an

* Britsh and For. Med. Review, III. 496, 498.

instrument would sooner perforate the coats of the canal, than admit of being forced through the closed and tortuous passage. In the present case, tubes, some of which were two feet in length, were introduced into the rectum, and water injected through them continually to facilitate their progress. But the more flexible tubes were bent into a coil in the rectum, and the more rigid ones were irresistibly stopped at the sigmoid flexure, and could not be further forced without danger of perforating the intestine, an accident well known to have followed injudicious and violent efforts.—*Boston Med. and Surg. Journal.*

